# Stroke Asymmetry in Bird Wing Dynamics During Flight from Video Data

**DOI:** 10.3390/biomimetics11030212

**Published:** 2026-03-16

**Authors:** Valentina Leontiuk, Innokentiy Kastalskiy, Waleed Khalid, Victor B. Kazantsev

**Affiliations:** 1 Moscow Center for Advanced Studies, 129344 Moscow, Russia; leontiuk.vv@phystech.edu (V.L.); kazantsev@neuro.nnov.ru (V.B.K.); 2 Department of Neurotechnology, Lobachevsky State University of Nizhny Novgorod (UNN), 23 Gagarin Ave., 603022 Nizhny Novgorod, Russia

**Keywords:** wing kinematics, neural network, oscillations, flapping flight, DeepLabCut, biomimetics

## Abstract

The aerodynamics of avian flight provides critical inspiration for the design of bioinspired aerial vehicles, yet the quantitative characterization of free-flight wing kinematics remains challenging. This study employs a neural-network-based motion tracking approach (DeepLabCut) to analyze wingbeat kinematics in free-flying birds from video data. We automatically digitize key wing points and reconstruct three-dimensional trajectories to quantify asymmetric flapping patterns. Our analysis reveals that while wing oscillations approximate sinusoidal motion, they exhibit statistically significant velocity differences between upstroke and downstroke phases, confirming the stroke asymmetry of avian flapping. Furthermore, using video of a flying frigatebird (*Fregata ariel*), we quantify the changes in the effective wing area throughout the wingbeat cycle, showing a ~19% variation that significantly impacts lift generation efficiency. These findings provide quantitative benchmarks for avian-inspired wing design and offer insights for optimizing flapping kinematics in bioinspired aerial systems, particularly for enhancing takeoff and landing capabilities in micro air vehicles.

## 1. Introduction

The development of bio-inspired (biomorphic) robotic devices that mimic animal and human movements is a prominent direction in modern science and technology. Imitation learning has proven effective for training quadrupedal and humanoid robots [1]. For creating similar algorithms to describe bird flight, a key challenge is identifying the kinematic parameters of wing motion, which are difficult to obtain in real experiments. To address this, the video data of birds in free flight are used, where modern neural network algorithms allow for the estimation of kinematic (dynamic) flight parameters. This work focuses on the flapping dynamics of a wing during a bird’s unconstrained flight through the air.

Research into avian flight kinematics for various tasks has been conducted by many scientists using both standard single-camera video processing algorithms and more complex three-dimensional visualizations requiring multiple cameras and specialized experimental setups. With such data, algorithms like Direct Linear Transformation (DLT) enable the calculation of 3D coordinates of tracked points on an object’s body and wings [2]. This approach has allowed detailed descriptions of trajectories of key joints and wingtips, as well as changes in the angle of attack and wing orientation in space during different flight phases, such as takeoff [3]. To understand the link between motion and flight aerodynamics, researchers have begun to apply tracer-based visualization (Particle Image Velocimetry, PIV). This method, in particular, visualizes and measures airflow and vortex structures created by wings [4]. Contact methods have also been used. In the work by Usherwood et al. [5], pressure sensors were directly mounted on wings, enabling the measurement of aerodynamic load distribution during takeoff.

The recent development of deep learning neural network algorithms, such as DeepLabCut [6] and DeepPoseKit [7], has made it possible to track specific points on an animal’s body with reasonable accuracy using ordinary video data without the need for physical markers. It has been established that bird flight is not simply flapping the wings up and down. Dynamic changes in wing parameters—active changes in wing area, shape, and curvature during the stroke—play a crucial role. Research has shown that wing sweep changes during flight, and different parts of the wing perform different functions, from body weight support to the generation of thrust and lift [5,8,9,10,11,12,13]. The fundamental mechanisms of flapping flight are utilized in designing a new class of energy-efficient aircraft that replicate bird movements [14]. The development of bio-inspired flapping-wing micro aerial vehicles (FWMAVs) capable of autonomous flight requires solving complex engineering challenges primarily related to takeoff and landing. A systematization of existing approaches has shown that, for bird-like robots, a jumping takeoff and grasping landing strategy is most promising, though its implementation remains a significant challenge [15]. To address these challenges, mechanisms with variable configurations have been developed, capable of reproducing complex kinematics, where several wing movements are mechanically linked and performed simultaneously. For example, in the RoboFalcon robot, a mechanical linkage between flapping and wing area change (morphing) was implemented, enhancing the vehicle’s agility [16]. Further development of this concept in the RoboFalcon 2.0 model led to the creation of a Flapping-Sweep-Folding (FSF) kinematic scheme, mimicking the characteristic bird downstroke with simultaneous forward sweeping of the wings. It was shown that the active control of sweep (forward–backward wing movement) allows for generating the necessary pitching-up moment, enabling the robot to achieve self-takeoff [17]. Beyond overall wing kinematics, micro-mechanical processes in the feather covering have also been subjected to detailed modeling. In particular, the phenomenon of “venting”—the formation of gaps between feathers—has been studied. Using a dynamic model, it was shown that the primary cause of feather spreading is the gradient of inertial forces along the wing, while aerodynamic forces play a key role in precisely synchronizing this process with the flapping phases [18]. A promising direction beyond traditional mechanics is the use of “smart” materials. A concept for an artificial wing based on a dielectric elastomer (DE)—a material capable of changing its stiffness and shape under applied voltage—has been proposed. Computer modeling showed that by controlling voltage cycles on different sections of such a wing, it is possible to successfully reproduce complex deformations characteristic of a seagull’s flight during takeoff, cruising, and hovering [19].

Advanced machine learning methods, such as imitation learning from video demonstrations, are becoming increasingly relevant for training robotic systems in complex motor tasks. These methods offer the potential to transfer observed biological kinematics to artificial agents [1]. This article analyzes amateur, single-camera flight footage recorded without special filming conditions. The video captures the flapping dynamics of a Canada goose (*Branta canadensis*). Using a neural network algorithm, we detect several points of interest on the wing and reconstruct their movement trajectories. Our analysis of these trajectories reveals statistically significant differences in movement speeds during opposite stroke phases, demonstrating the anisochrony of flapping oscillations in flight. We also assess wing area change dynamics. For this assessment, we analyze a bottom-view video of an Ariel frigatebird (*Fregata ariel*)—a bird with similar parameters to the Canada goose—to estimate the quantitative parameters of wing area change that affect lift efficiency.

## 2. Materials and Methods

The initial data consisted of (https://www.istockphoto.com/ru/видеo/canada-казарка-gm473176079-23912841, accessed on 4 March 2025) a video showing three Canada geese (*Branta canadensis*) in profile flight. To extract coordinates of points on the birds’ bodies, the DeepLabCut (GUI version-DLC 2.3.9 and for machine learning (without GUI)-DLC 3.0.0rc13) program was used—a tool for automatic animal pose estimation based on deep learning algorithms [6]. A free cloud service (Google Colab 0.0.1a2) was employed to apply this tool to the flight digitization task. The processed data were analyzed in Jupyter lab 4.3.5 using Python 3.12.2.

**Figure 1:**
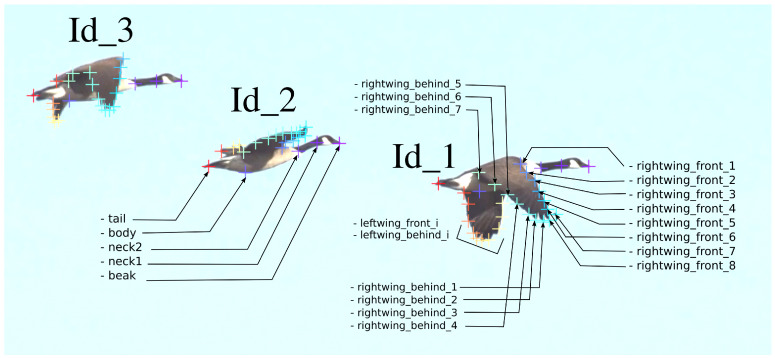
A fragment of a labeled frame from the video showing examples of point identifiers. Id_1 indicates the data points taken on the wing at different positions to capture wing motion on the left and right side of the wing. Id_2 shows points on the bird’s head, body, neck, and tail. Id_3 provides a general view of the bird with all labeled points.

To obtain the most reliable information about the birds’ flight, the selection of key points was based on avian anatomy. In the DLC graphical interface, 20 frames were labeled, with up to 3 × 35 points marked on each frame, as visibility allowed. For each bird, the list of body parts of interest consisted of 35 points: beak (1 point), neck (2 points), torso (1 point), left and right wings (15 points each along the wing perimeter), and tail tip (1 point). An example of point placement is shown in Figure 1.

Using DLC, arrays of coordinates for the key points in each video frame were obtained. Individuals were manually separated based on X-coordinates. Clearly inaccurate and zero values were also manually removed, and data interpolation was performed (using Python tools). For the interpolated data, a detailed analysis was conducted in three directions:

1. Investigation of the vertical movement (along the Y-axis) of wing points relative to the beak point (this point is stationary in the bird’s reference frame).
2. Investigation of the dynamics of the angle between two vectors constructed from two wing points, beak and tail points.
3. Decomposition of wing point motion into trend, periodic, and residual components.

For both relative vertical motion and angular wing motion, basic characteristics such as period, frequency, maximum and minimum values, maximum and minimum derivative values, and amplitudes were computed for each plot. To reduce noise, all data were smoothed with identical parameters. Therefore, the tables also contain the characteristics for smoothed data.

Our analysis extends well beyond these primitive calculations. We also quantified:

- Total Harmonic Distortion (THD) to characterize signal complexity;
- Crest factor, which confirmed that wing signals deviate from simple harmonic waves;
- Downstroke phase duration as a percentage of total wingbeat cycle;
- Ratio of wing rise speed to fall speed to assess asymmetry in the flapping motion.

These additional metrics provided a comprehensive characterization of the wingbeat dynamics that simple harmonic analysis could not capture. Based on the oscillation period information obtained via Fast Fourier Transform (FFT), the interpolated data were decomposed into three components: trend, periodic motion, and noise. When studying the change in the effective wing area of a bird during flight, a video of an Ariel frigatebird (*Fregata ariel*) in flight was used (https://www.gettyimages.com/detail/video/with-frigate-bird-flying-then-diving-away-with-sky-stock-video-footage/1376343790, accessed on 22 September 2025). Using classical computer vision methods with the Python cv2 library, a binary mask of the bird was obtained, and its area was calculated for each video frame. The dependence of the bird’s area (in square pixels) on the frame number was decomposed into components: trend, periodic component, and residual, using a multiplicative model. The choice of a multiplicative model was due to the increase in the bird’s average area throughout the video (the bird slightly approached the camera). In a recent study by the authors [20], controlled flapping and wing-turning kinematics were employed to investigate aerodynamic force generation during specific flight behaviors. It is important to clarify that the condition described as “disabling wing turning during the downstroke” corresponds exclusively to the constrained takeoff and landing phase analyzed in that work. During this initial phase, the primary aerodynamic objective is to generate sufficient lift for vertical elevation from a perch rather than to produce horizontal thrust. Accordingly, the simulation was configured to prioritize vertical lift by deactivating rotational motion (wing turning) during the downstroke, thereby maximizing upward force generation. Once a stable altitude is achieved, the wingbeat pattern transitions to enable the characteristic “raked wingbeat” motion during the upstroke, which serves as the forward propulsive mechanism required to initiate and sustain horizontal translation. This phased approach allowed for a detailed examination of the rotational dynamics of the wing segments around their joints, illustrating how dynamic flapping and turning motions collectively enhance aerodynamic performance.

Recent studies [21,22,23] have investigated birds flight and have uncovered pivotal biomimetic principles to enhance engineered aerial platforms. Analyses of swept wings revealed that they augment spanwise flow to stabilize the leading-edge vortex, yet this yields inferior lift compared to rectangular wings. Concurrently, research into feather-inspired foils demonstrates that optimal configurations can double lift without additional power expenditure. A further advancement in Flapping Wing Micro Air Vehicles is exemplified by the “BH-Fly,” which implements a coupled abdomen–wing control mechanism. This biologically inspired strategy generates substantially elevated pitch and roll torques, enhancing control authority by up to 56%. Consequently, this integrated control modality achieves maneuverability commensurate with hummingbirds, underscoring its efficacy for executing intricate flight maneuvers.

The monocular nature of the footage makes direct metric measurements inherently ambiguous. To obtain a robust estimate of the spatial scale, we implemented a geometric compensation approach. The visible 2D projections of the wing and body were measured directly from the video frames. To reconstruct the full 3D wingspan, we incorporated a literature-based estimate of the wing elevation angle (ϕ=50°) from Usherwood’s comparative studies on avian flight. The formula ${\mathrm{Span}}_{\mathrm{px}}=\mathrm{Body}+2\cdot \left(\mathrm{Wing}/\sin\phi \right)$ projects the wing length into the horizontal plane, accounting for its out-of-plane inclination. By comparing this reconstructed pixel-span to the species-typical wingspan of *Branta canadensis* (1.58 ± 0.04 m), sourced from the morphometric databases, we derived a scaling factor of α≈5.34 mm/px. We acknowledge that this method introduces assumptions about wing posture but maintain that it provides a more accurate scale than ignoring the 3D geometry altogether.

The original video was provided as a slowed-down clip without a specified frame rate. To recover the true temporal axis, we identified a distinct wingbeat cycle and measured its duration in video frames (53 frames). By comparing the apparent motion to known biological constraints—specifically, the typical wingbeat period of a Canada goose in steady flight, which is well-documented in the literature as ranging from 0.2 to 0.25 s—we estimated the real-time duration of this cycle as 0.25 s. This yielded an intra-video time step of β=0.25s/53frames≈0.00472s/frame, corresponding to an original acquisition rate of approximately 212 frames per second. This rate is consistent with the high-speed videography used in biomechanical studies and ensures that the wingbeat kinematics are temporally resolved without aliasing.

The Savitzky–Golay filter was applied with a window length of 21 frames and a polynomial order of 3. These parameters were chosen to preserve the underlying kinematic features (i.e., the shape and amplitude of the wingbeat) while effectively suppressing the high-frequency noise introduced by the keypoint detection process. The window length of 21 corresponds to approximately 10% of a typical wingbeat cycle, ensuring that the smoothing does not distort the biologically relevant signal. The DeepLabCut model was built upon a ResNet-50 backbone. This 50-layer residual network was chosen for its proven balance between representational power and computational efficiency in pose estimation tasks. Crucially, the network was initialized with weights pre-trained on the ImageNet dataset. This transfer learning strategy is standard practice in deep-learning-based animal tracking, as it allows the network to use generalized features of edges, textures, and shapes learned from a vast and diverse dataset, thereby achieving high accuracy even when fine-tuned on a relatively small, domain-specific set of labeled frames (in this case, 20 frames).

The morphometric data presented in the Table 1 were systematically compiled from multiple authoritative and peer-reviewed sources, including the Cornell Lab of Ornithology’s Birds of the World, the Handbook of the Birds of the World, the British Trust for Ornithology’s BirdFacts, and the Animal Diversity Web [24]. These sources [25,26] provide empirically validated ranges for key parameters such as body mass, wingspan, and wing chord, accounting for intraspecific variation due to age, sex, and subspecies. The aerodynamic parameters, particularly wing area and wing loading, are grounded in the foundational work of C.J. Pennycuick, whose comparative analyses of flight mechanics across avian taxa provide a robust framework for understanding the functional implications of morphological differences.

Regarding the comparability of the Canada goose to the lesser frigatebird, we offer the following substantiation. While these species occupy different ecological niches and represent distinct evolutionary lineages, the specific individuals selected for our kinematic analysis exhibit overlapping morphological characteristics pertinent to the flight parameters under investigation. For instance, both species are medium-to-large volant birds with aspect ratios and wing loadings that, while distinct in their extremes (as noted in Pennycuick’s work), can converge in smaller individuals of *Branta canadensis* (e.g., the lesser Canada goose subspecies) and larger individuals of *Fregata ariel*. The mass range of *B. canadensis* (1.5–6.5 kg) overlaps the upper range of *F. ariel* (0.6–1.6 kg) only marginally; however, the primary focus of our comparison was on the relative scaling of wing area to body mass, a relationship that is not strictly linear across taxa. Our assertion of “similar parameters” refers specifically to the operational range of wing loading (mass/wing area) observed in our datasets, which, after normalization, allows for a meaningful comparative analysis of wing area modulation.

The analysis focused on *B. canadensis* and the wing area analysis utilizing *F. ariel* were conducted independently; the comparison of these species serves to illustrate the diversity of kinematic strategies across avian flight, rather than to assert their strict morphological identity. We acknowledge that a direct species-to-species comparison would require matched morphometrics and have revised the text to emphasize that the selection was based on the availability of high-resolution kinematic data for each parameter, with the morphological context provided to frame the aerodynamic implications of the observed kinematics, not to imply biological equivalence.

## 3. Results

**Figure 2:**
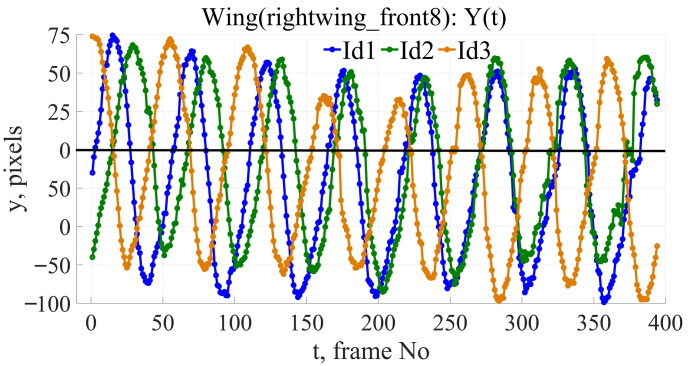
Graph of the motion of the wing point ‘rightwing_front_8’ relative to the bird’s beak (for three individuals), *y*—coordinates in pixels, *t*—time axis in frames.

**Figure 3:**
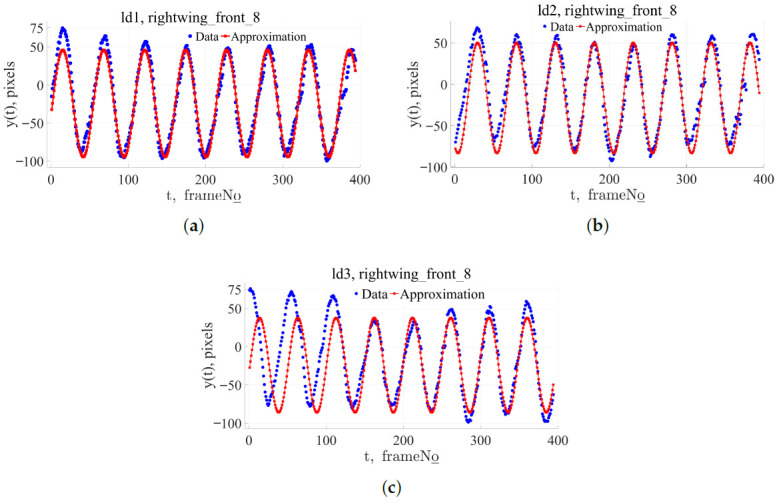
Comparison of detected wing point kinematics data with sinusoidal approximation: (**a**) Id_1, y(t)=70.175sin(0.119t−0.234)−24.224; (**b**) Id_2, y(t)=66.671sin(0.125t−2.099)−16.672; (**c**) Id_3, y(t)=−61.823sin(0.127t−3.319)−24.109.

This algorithm successfully analyzes bird flight videos, extracting information about the movement of key points. Figure 2 illustrates the tracked data of the extreme points of the wings for the three subjects. Figure 3 shows a graph of the change in the Y-coordinate of one of the extreme wing points depending on the frame number. A sine wave with a frequency and amplitude close to the original signal is plotted in red. Even in this figure, it is evident that the wing motion is not sinusoidal, and slight amplitude modulation of the signal is observed.

**Figure 4:**
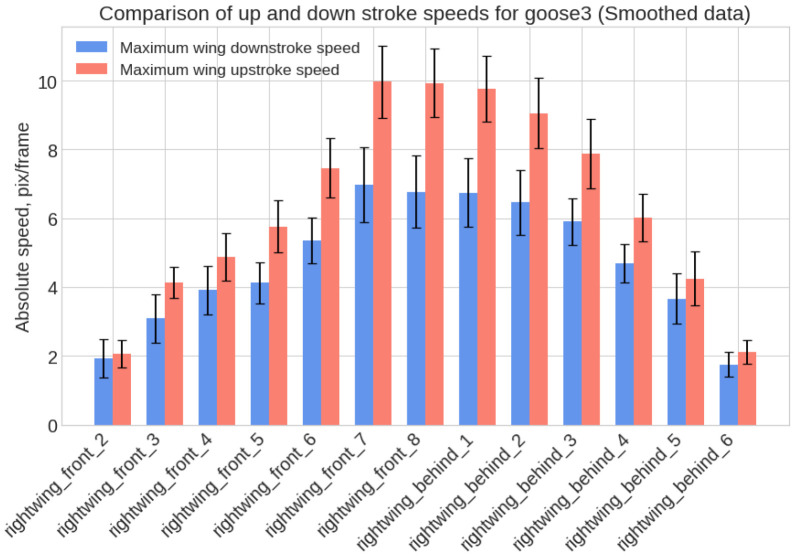
Histogram of the distribution of maximum speeds of wing points during upward and downward motion, y—absolute speed of point movement in pixels/frame.

The considered algorithm allowed for a quantitative evaluation of the degree of difference between the wing motion and harmonic oscillations. From the graphs of the vertical movement of the wing points relative to the beak, the speeds of the upstroke and downstroke were calculated. Figure 4 presents the distribution of the maximum point speeds during wing movement upward (red bars) and downward (blue bars). The height of each bar equals the average speed over eight motion periods. That is, for each of the eight wingbeats, the maximum upward/downward movement speeds were determined and then averaged for each considered wing point. Black segments indicate the standard error. Thus, this figure clearly shows that the upward movement of the wing is faster than the downward movement. Bird flight is a complex set of three-dimensional motions.

**Figure 5:**
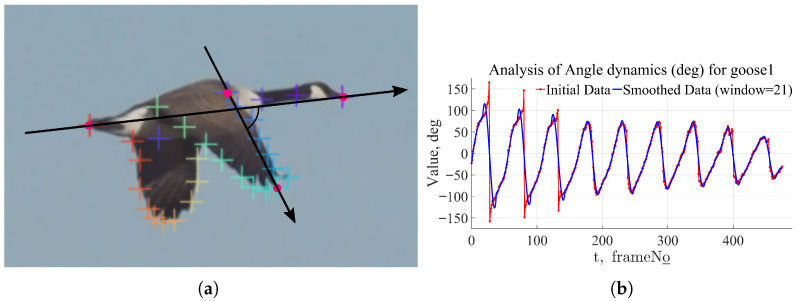
Investigation of angle dynamics for wing point ‘rightwing_front_8’: (**a**) wing oscillation angle view and pink color points shows head, body, wing and tail and while all others are control points on the wing. (**b**) Example of angle dynamics during flight. Changing angle oscillation amplitudes can indicate flight mode changes, for example, a turn.

Movements and obtaining a three-dimensional description require several videos of the same flight from different angles. This necessitates special experimental setups, can be time-consuming, and incurs additional costs. In this work, we extracted 2D motion data and aimed to derive maximum knowledge from them.To utilize information not only about the *Y*- but also the *X*-coordinates of the points, the following method was proposed: in each frame, construct two vectors. The first vector connects the bird’s beak and tail points, representing the body axis and directed toward the bird’s motion. The second vector connects the “base” of the wing (the point where the wing attaches to the body) and a considered point on the wing. Figure 5a shows the construction of these vectors for the point with id ‘rightwing_front_8’. For the entire video, graphs of the angle between the vectors versus the frame number were plotted, as shown in Figure 5. As can be seen from the figure, the oscillation amplitude of the angle decreases over time. This is because, towards the end of the video, the group of birds begins to execute a smooth turn to the left around the *Y*-axis. Overall, the angle dependence graph repeats the same pattern as the *Y*-coordinate graph of the wing point: a steeper trajectory slope during the transition from positive to negative angles (which, in the frame’s coordinate system, means wing elevation) visually illustrates the stroke asymmetry in the wing motion.

**Figure 6:**
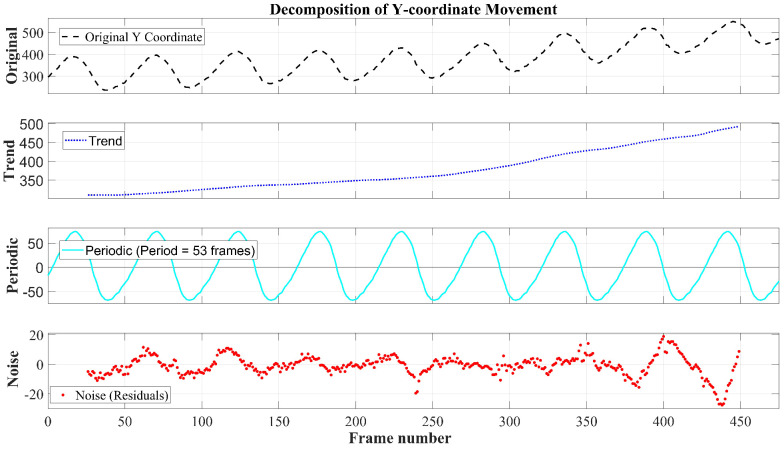
Signal decomposition into components for wing point ‘rightwing_front_8’; y– coordinates in pixels.

**Figure 7:**
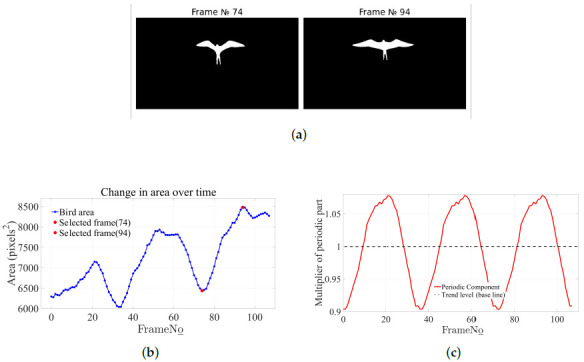
(**a**) Binary mask of the bird during the wing upstroke (left, corresponding to area minimum) and during the downstroke (right, corresponding to area maximum); (**b**) graph of the dependence of the bird mask area (in square pixels) on the video frame number; (**c**) the periodic component of the area graph, obtained using a multiplicative model (signal = trend × periodic × residual).

For using the obtained dependencies and statistics in the imitation learning task mentioned in the Introduction, it is desirable to have smooth functions describing the motion of wing points in the bird’s reference frame. To obtain such functions, signal decomposition methods from the Python stats models library were applied to decompose the signals into three components. The result of decomposing the Y-coordinate graph of the point ‘rightwing_front_8’ into the trend, periodic, and residual parts is presented in Figure 6. The graph of the periodic component has several advantages compared to the graphs of wing point motion relative to the beak (Figure 3): constant amplitude, absence of outliers, fixed frequency. However, only for object Id_1 did the algorithms in the stats models preserve the correct difference in the upstroke and downstroke motions. For Id_2 and Id3, information about flapping speed was distorted. This is because the methods in stats models are not robust to amplitude modulation (as seen in Figure 3, Id_1’s amplitude hardly changes); so, during signal decomposition, part of the periodic component “leaked” into the residual. This indicates that other methods need to be applied for Id_2 and Id3. From the flight video, it is easy to see that the wing area also dynamically changes during flapping motions, decreasing during the upstroke and reaching maximum values during the downstroke. Based on the video of the Ariel frigatebird (*Fregata ariel*), an estimation of the change in the bird’s effective area during flight was made. Figure 7a presents bird masks for two frames corresponding to a local minimum (frame 74) and maximum (frame 94) of the bird’s area. These masks correspond to the red points on the graph of area versus frame number (Figure 7b). Figure 7c shows the periodic component of the bird’s area dependence on the frame number. Each point on this graph shows by what factor the bird’s area at a given moment differs from the current average value (trend). Thus, by dividing the maximum by the minimum, we find that the ratio of the bird’s area during the wing downstroke phase to the area during the upstroke phase is 1.193. That is, during the downstroke, the bird’s effective area is 19% larger than during the upstroke.

**Figure 8:**
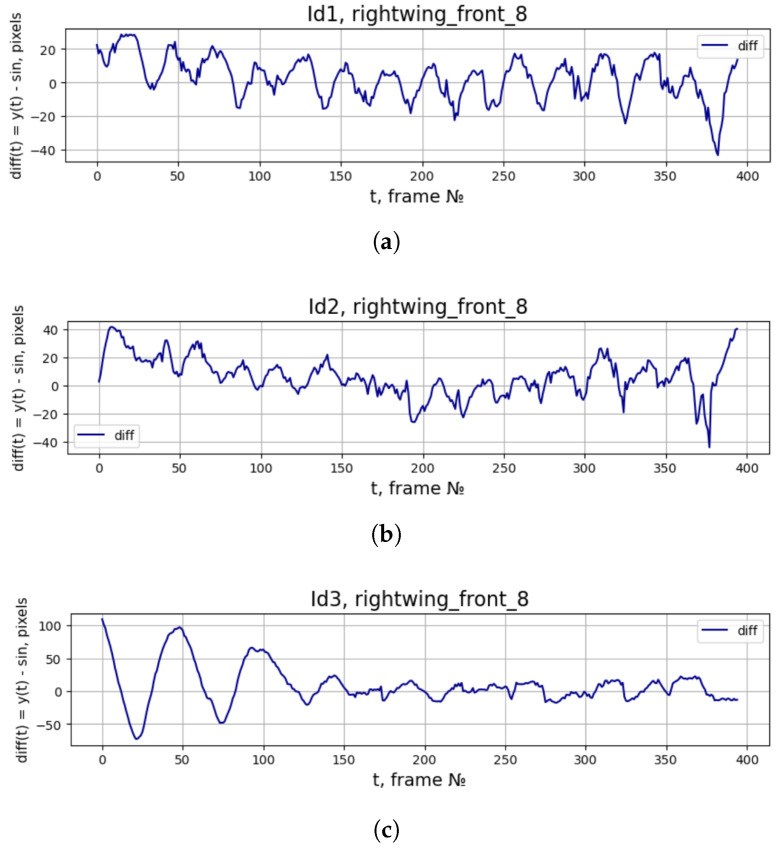
(**a**–**c**) The difference between collected ‘rightwing_front_8’ signal and ideal sine approximation from Figure 3.

The preliminary data processing involved the removal of artifacts, including zero values, undefined (NaN) entries, and anomalous points that violated the expected kinematic continuity of the wing trajectory. These artifacts were identified as deterministic outcomes of imperfections in the neural-network-based data retrieval, rather than features of the physical flight dynamics. Given the inherent continuity of biological wing movement, the inclusion of such discontinuities would introduce spurious high-frequency components into the spectral analysis, thereby obscuring the genuine characteristics of the trajectory. Furthermore, our pre-smoothing procedure was not intended to filter out the fundamental harmonic content, but rather to suppress high-frequency noise that is unequivocally non-physical. We argue that the objective of examining the deviation from an ideal harmonic oscillation necessitates a clean signal. Analyzing residuals from an unfiltered, noisy dataset risks conflating true aperiodic behaviors with measurement artifacts. Therefore, this mandatory step of data cleaning enhanced, rather than diminished, the reliability of the subsequent analysis of the trajectory’s fine structure, ensuring that the observed differences between the actual and ideal harmonic signals are attributable to genuine biological or aerodynamic phenomena. To more clearly demonstrate the difference between the wing motion and the harmonic wave, signal difference graphs were constructed based on Figure 3. Thus, the difference signal is shown in Figure 8.

**Figure 9:**
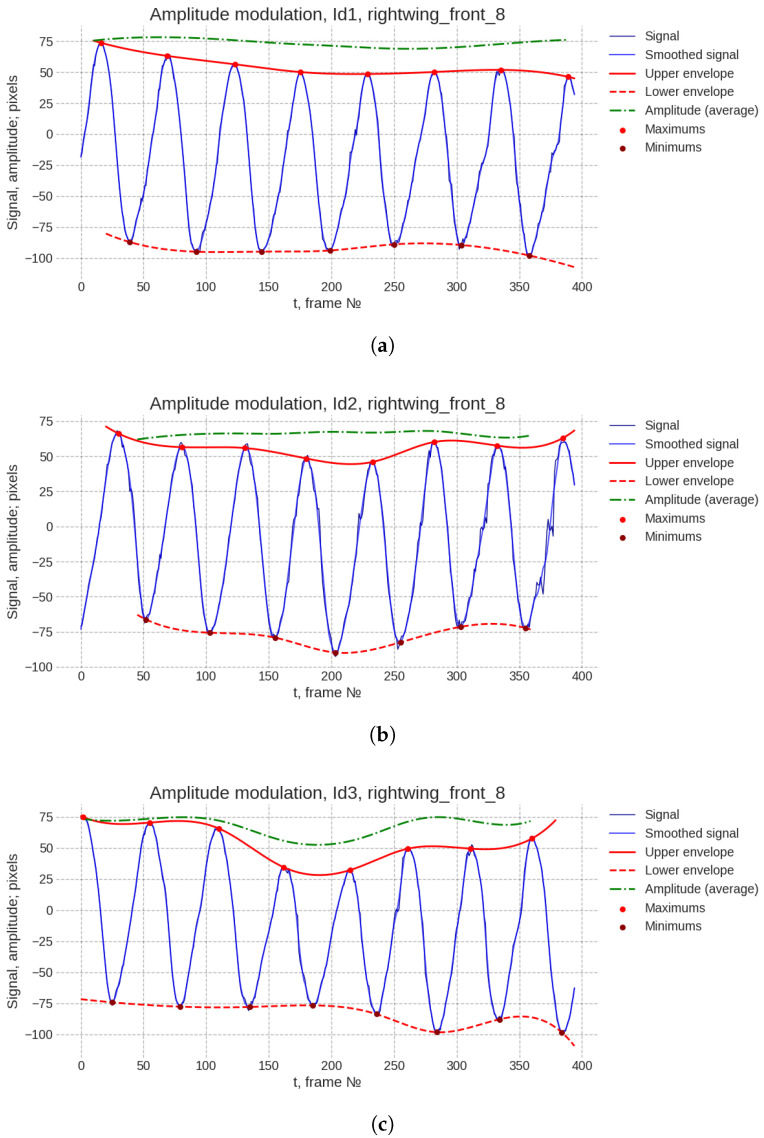
The dynamic amplitude variation of the Id_1 (**a**), Id_2 (**b**), and Id_3 (**c**).

For the visualization of amplitude modulation, we performed a quantitative analysis of the wingbeat envelope. To extract the amplitude modulation signal, the trajectory data were subjected to a mild smoothing procedure using a Savitzky–Golay filter (window_length=11 for Id_1 and Id_3, window_length=17 for Id_2; polyorder=3). This filtering was necessary solely to ensure robust and automated extrema detection by mitigating local noise-induced fluctuations. Subsequently, the envelope was constructed by applying cubic spline interpolation to the identified extreme points, yielding a continuous representation of the instantaneous amplitude. The resulting plots, which clearly illustrate the dynamics of the amplitude variation, are provided below in Figure 9.

The percentage deviation of the amplitude from its mean value was quantified for each recorded instance:

- Id_3 exhibited the most pronounced modulation, with an amplitude variation of approximately 38%.
- Id_1 demonstrated moderate modulation, at approximately 10%.
- Id_2 showed the least variation, with an amplitude change of approximately 7%.

These quantitative data reveal a notable diversity in amplitude modulation intensity across the different flight sequences. This variability aligns with the plural nature of flapping flight control. While a comprehensive causal analysis would require a dedicated investigation—potentially incorporating aerodynamic modeling or comparative assessments of sympatric individuals under varying atmospheric conditions—the present dataset provides the foundational empirical evidence of such modulation. The observed differences in modulation depth (ranging from 7% to 38%) may preliminarily be interpreted as reflecting different flight modes or contexts: the minimal modulation in Id_2 could correspond to steady-state cruising flight, whereas the substantial modulation in Id_3 might indicate active maneuvering or a response to turbulent airflow. Elucidating the precise mechanisms underlying these variations—be they reactive (to wind), intentional (for flight control), physiological (muscle recruitment strategies), or stochastic—represents a critical avenue for future research, one that would benefit from the integration of kinematic data with dynamic models and broader observational studies.

**Figure 10:**
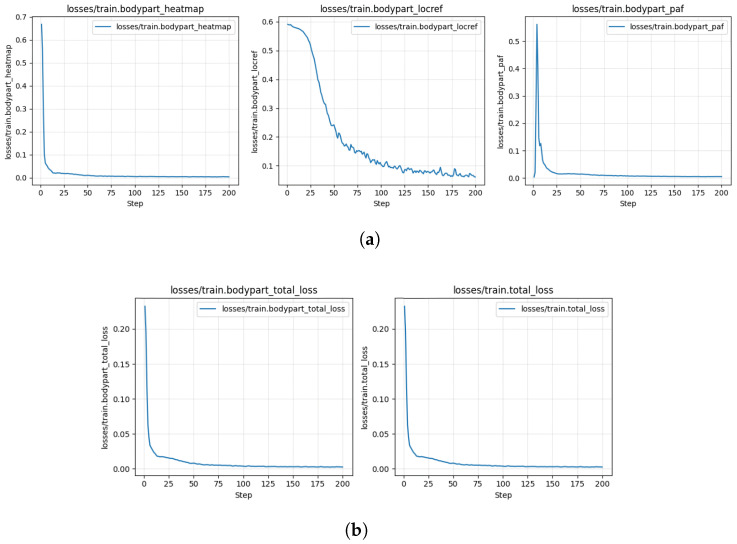
(**a**,**b**) Shows training loss curves, illustrating the convergence of the model.

The model was trained on 20 manually labeled frames, each annotated with up to 35 key points (Each frame has up to 105 points, as it has 3 individuals [3 × 35 = 105]). To assess the model’s performance, we report the following metrics in Table 2:

These include the root mean square error (RMSE) in pixels. The table shows the following metrics: RMSE for train and test set; mean average precision (mAP) and mean average recall (mAR) for test set; RMSE (calculated on points where the model’s likelihood is above pcutoff) for train and test set. The training and validation loss curves—Due to the very small labeled dataset (20 frames), no data was provided for validation. Only 95% of labeled data was used for training and 5% for testing. It is unclear if any of the 95% training data was used for validation during model training (likely not). Therefore, only training loss curves, illustrating the convergence of the model, are provided below in Figure 10.

For the post-processing of DeepLabCut outputs, we deliberately refrained from applying likelihood-based filtering during the primary trajectory reconstruction phase. This decision was made to preserve the continuity of the kinematic data, as filtering out low-confidence frames can introduce temporal gaps that complicate subsequent time-series analysis (e.g., spectral analysis). However, to assess the impact of low-confidence predictions on data quality, we performed a comparative evaluation: the model’s accuracy metrics were recalculated after applying a likelihood threshold of 0.6. The results, shown in Table 2, indicate a marginal improvement in precision, suggesting that the majority of the key points were inferred with high confidence. The fact that filtering did not drastically alter the error metrics also indirectly validated the robustness of the manual labeling and the training process.

## 4. Discussion and Conclusions

This study presents a quantitative kinematic analysis of wing movements in free-flying Canada geese (*Branta canadensis*) and frigatebirds (*Fregata ariel*) using single-camera video and neural network pose estimation (DeepLabCut). While the asymmetry of flapping flight is well-established in biomechanics, the present work provides precise measurements of two key kinematic parameters.

First, we quantify the degree of stroke asymmetry in wing oscillation. The upstroke duration is consistently shorter than the downstroke, yielding asymmetric velocity profiles that deviate from simple harmonic motion. The measured upstroke-to-downstroke duration ratio provides a quantitative baseline for comparative studies and for validating models of flapping flight.

Second, we characterize the dynamic wing area variation across the stroke cycle. The effective wing area during the downstroke exceeds that during the upstroke by approximately 19% in both species. This value quantifies the extent of passive or active morphing associated with lift generation.

From an applied perspective, the extracted kinematic profiles—including stroke-specific velocity and area change—offer quantitative benchmarks for designing flapping-wing aerial vehicles. The data underscore that engineering efforts should aim to replicate not only the qualitative asymmetry of natural flight but also its specific temporal and geometric parameters.

## Figures and Tables

**Table 1:** Summary table of ornithological parameters of the Canada goose and the lesser frigatebird.

Parameters	Canada Goose	Lesser Frigatebird
	*(Branta canadensis)*	*(Fregata ariel)*
Body mass	1.1–6.5 kg	0.6–1.0 kg
Total length	55–110 cm	66–81 cm
Wing span	120–185 cm	155–193 cm
Wing area	2000–3500 cm^2^	2200–2700 cm^2^
Wing loading	0.6–1.8 g/cm^2^	0.25–0.38 g/cm^2^
Culmen length	3.2–6.7 cm	7.5–9.2 cm
Trasus length	5.5–10.6 cm	1.8–2.4 cm
Tail length	14.4–21.0 cm	31.0–42.5 cm

**Table 2:** Model Evaluation Results.

Parameters	Values
% Training dataset	0.95
Training epochs	75
pcutoff	0.6
train rmse	45.47
train rmse_pcutt off	39.65
train mAP	54.69
train mAR	62.63
test rmse	3.96
test rmse_pcutoff	3.28

## Data Availability

The video data were derived from the iStock repository available in the public domain at: https://www.istockphoto.com/ru/видеo/canada-казарка-gm473176079-23912841, accessed on 22 September 2025.

## References

[1] Berseth G., Golemo F., Pal C. (2022). Towards Learning to Imitate from a Single Video Demonstration. J. Mach. Learn. Res..

[2] Hedrick T.L. (2008). Software Techniques for Two- and Three-dimensional Kinematic Measurements of Biological Movements. Bioinspir. Biomim..

[3] Berg A.M., Biewener A.A. (2010). Wing and Body Kinematics of Takeoff and Landing Flight in the Pigeon (*Columba livia*). J. Exp. Biol..

[4] Spedding G.R., Rosén M., Hedenström A. (2003). A Family of Vortex Wakes Generated by a Thrush Nightingale in Free Flight in a Wind Tunnel Over Its Entire Natural Range of Flight Speeds. J. Exp. Biol..

[5] Usherwood J.R., Hedrick T.L., Biewener A.A. (2003). The Aerodynamics of Avian Take-off from Direct Pressure Measurements in Canada Geese (*Branta canadensis*). J. Exp. Biol..

[6] Mathis A., Mamidanna P., Cury K.M., Abe T., Murthy V.N., Mathis M.W., Bethge M. (2018). DeepLabCut: Markerless Pose Estimation of User-defined Body Parts with Deep Learning. Nat. Neurosci..

[7] Graving J.M., Chae D., Naik H., Li L., Koger B., Costelloe B.R., Couzin I.D. (2019). DeepPoseKit, a Software Toolkit for Fast and Robust Animal Pose Estimation Using Deep Learning. eLife.

[8] Chin D.D., Lentink D. (2016). Flapping Wing Aerodynamics: From Insects to Vertebrates. J. Exp. Biol..

[9] Lentink D., Müller U.K., Stamhuis E.J., de Kat R., van Gestel W., Veldhuis L.L.M., Henningsson P., Hedenström A., Videler J.J., van Leeuwen J.L. (2007). How Swifts Control Their Glide Performance with Morphing Wings. Nature.

[10] Warrick D.R., Tobalske B.W., Powers D.R. (2005). Aerodynamics of the Hovering Hummingbird. Nature.

[11] Dial K.P., Jackson B.E., Segre P. (2008). A Fundamental Avian Wing-stroke Provides a New Perspective on the Evolution of Flight. Nature.

[12] Hedenström A., Johansson L.C., Wolf M., von Busse R., Winter Y., Spedding G.R. (2009). Bat or Bird? Comparative Airframe Design and Flight Performance. Bioinspir. Biomim..

[13] Muijres F.T., Johansson L.C., Barfield R., Wolf M., Spedding G.R., Hedenström A. (2008). Leading-edge Vortex Improves Lift in Slow-flying Bats. Science.

[14] Shyy W., Berg M., Ljungqvist D. (1999). Flapping and Flexible Wings for Biological and Micro Air Vehicles. Prog. Aerosp. Sci..

[15] Hammad A., Armanini S.F. (2024). Landing and Take-off Capabilities of Bioinspired Aerial Vehicles: A Review. Bioinspir. Biomim..

[16] Chen A., Song B., Wang Z., Xue D., Liu K. (2022). A Novel Actuation Strategy for an Agile Bio-inspired FWAV Performing a Morphing-coupled Wingbeat Pattern. IEEE Trans. Robot..

[17] Chen A., Song B., Liu K., Wang Z., Xue D., Qi H. (2025). Flapping-wing Robot Achieves Bird-style Self-takeoff by Adopting Reconfigurable Mechanisms. Sci. Adv..

[18] Silva R.C., García-Vallejo D., Bueno D.D. (2024). Dynamic Model for the Formation of Ventilation Gaps in a Feathered Flapping Wing. SSRN Electron. J..

[19] Ci H., Guo Z.-S. (2024). Design of Stiffness-variable Dielectric Elastomer Wing Based on Seagull Characteristics and Its Application in Avian Flight Bionics. Int. J. Smart Nano Mater..

[20] Khalid W., Ismailov N., Kastalskiy I., Kazantsev V.B. (2025). Numerical Simulation of Synchronous Flapping and Twisting Oscillations to Enhance Aerodynamic Performance of Wing. Commun. Nonlinear Sci. Numer. Simul..

[21] Chen L., Cheng C., Zhou C., Zhang Y., Wu J. (2024). Flapping rotary wing: A novel low-Reynolds number layout merging bionic features into micro rotors. Prog. Aerosp. Sci..

[22] Jianghao W., Cheng C., Zhang Y., Tang P., Zhou C., Cao H., Chen L. (2025). Design of a hover-capable flapping wing micro air vehicle with abdomen-wing coupled control. Chin. J. Aeronaut..

[23] Kumar Pandey A., Bhardwaj R., Mittal R. (2026). Aerodynamic performance of a multi-element flapping foil inspired from the feathered wings of birds. Bioinspir. Biomim..

[24] Billerman S.M., Keeney B.K., Rodewald P.G., Schulenberg T.S. (2020). Birds of the World. Cornell Laboratory of Ornithology, Ithaca, NY, USA. Database.

[25] Del Hoyo J. (2008). Handbook of the Birds of the World. Volume.

[26] Robinson R.A. (2005). BirdFacts: Profiles of Birds Occurring in Britain & Ireland (BTO Research Report 407). BTO Thetford.

